# *Ex-vivo* tolerogenic F4/80^+^ antigen-presenting cells (APC) induce efferent CD8^+^ regulatory T cell-dependent suppression of experimental autoimmune uveitis

**DOI:** 10.1111/cei.12243

**Published:** 2014-03-06

**Authors:** S-M Hsu, R Mathew, A W Taylor, J Stein-Streilein

**Affiliations:** * Schepens Eye Research Institute, Department of Ophthalmology, Harvard Medical SchoolBoston, MA, USA; ‡ Department of Ophthalmology, Boston University School of MedicineBoston, MA, USA; † Department of Ophthalmology, National Cheng-Kung University HospitalTainan City, Taiwan

**Keywords:** ACAID, autoimmunity, CD8^+^ T_reg_, EAU, tolerogenic APC

## Abstract

It is known that inoculation of antigen into the anterior chamber (a.c.) of a mouse eye induces a.c.-associated immune deviation (ACAID), which is mediated in part by antigen-specific local and peripheral tolerance to the inciting antigen. ACAID can also be induced *in vivo* by intravenous (i.v.) inoculation of *ex-vivo*-generated tolerogenic antigen-presenting cells (TolAPC). The purpose of this study was to test if *in-vitro*-generated retinal antigen-pulsed TolAPC suppressed established experimental autoimmune uveitis (EAU). Retinal antigen-pulsed TolAPC were injected i.v. into mice 7 days post-induction of EAU. We observed that retinal antigen-pulsed TolAPC suppressed the incidence and severity of the clinical expression of EAU and reduced the expression of associated inflammatory cytokines. Moreover, extract of whole retina efficiently replaced interphotoreceptor retinoid-binding protein (IRBP) in the preparation of TolAPC used to induce tolerance in EAU mice. Finally, the suppression of EAU could be transferred to a new set of EAU mice with CD8^+^ but not with CD4^+^regulatory T cells (T_reg_). Retinal antigen-pulsed TolAPC suppressed ongoing EAU by inducing CD8^+^ T_reg_ cells that, in turn, suppressed the effector activity of the IRBP-specific T cells and altered the clinical symptoms of autoimmune inflammation in the eye. The ability to use retinal extract for the antigen raises the possibility that retinal extract could be used to produce autologous TolAPC and then used as therapy in human uveitis.

## Introduction

The eye is the prototype for the study of immune-privileged mechanisms. Because ocular antigens are not sequestered from recognition by the immune system [1], multiple layers of immune regulation exist, both locally and peripherally, to preserve the visual axis. Ocular-induced regulation of immune responses suppress inflammation and the adaptive immune response, in part, by generating antigen-specific regulatory T cells (T_reg_) that contribute to both local and peripheral tolerance [2]. In addition, activated T cells specific for ocular antigens that are able to cross the structural barriers of the eye meet multiple immunosuppressive mechanisms to prevent them from finding their targets and inducing inflammation within the eye [2–5].

In spite of all the overlapping immunoregulatory mechanisms that exist, uveitis occurs in approximately 0·2% of the US population [6], with autoimmunity contributing to approximately 50% of the aetiology. While 0·2% of the population is not classified as an orphan disease, the National Institute of Health (NIH) considers it a rare disease. Autoimmune uveitis is a sight-threatening inflammatory disorder that affects all ages, and is a significant cause of visual loss [7]. Each year 17·6% of active uveitis patients experience a transient or permanent loss of vision, and 12·5% will develop glaucoma [8]. Uveitis is also associated with several systemic diseases, including arthritis [9]. The medical community predominantly treats the clinical symptoms of uveitis with corticosteroids, with increasing prescriptions for biologicals such as anti-tumour necrosis factor (TNF)-α compounds [10]. A more specific treatment and restoration of the immune homeostasis would be a welcome treatment for uveitis.

Experimental autoimmune uveitis (EAU) is a disease of the neural retina that is induced by immunization of rodents (mice or rats) with retinal antigens. Generally, the antigen is introduced in concert with strong adjuvant and pertussis toxin injections to overcome natural immune resistance [11]. Because the use of strong adjuvants would interfere with the induction of tolerance, we used a well-established adoptive transfer model of EAU, which induces EAU by transferring interphotoreceptor retinoid-binding protein (IRBP)-primed T cells to naive recipients in the absence of adjuvants [7]. The purpose of this study was to determine if restoration of aspects of immune privilege in a mouse with EAU would interfere with and suppress the progression of the uveitis. More specifically, we wanted to know if we could induce suppression of the immune response to the inciting retinal antigen by inducing tolerance by the transfer of *ex-vivo*-generated TolAPC to EAU mice.

## Materials and methods

### Animals

C57BL/6J (B6) female (8–12 weeks old) mice were purchased from Jackson Laboratory (Bar Harbor, ME, USA) and used for all experiments. All animals were treated humanely and in accordance with the guidelines of the NIH office of Laboratory Animal Welfare; the protocols were approved by the Schepens Institutional Animal Care and Use Committee. All experiments were conducted in accordance with the Association for Research in Vision and Ophthalmology (ARVO) Statement for the Use of Animals in Ophthalmic and Vision Research.

### Reagents

Cells were cultured in serum-free medium (SFM), consisting of RPMI-1640 (Lonza, Walkersville, MD, USA), 10 mM HEPES, 0·1 mM non-essential amino acids, 1 mM sodium pyruvate, 100 U/ml penicillin and 100 μg/ml streptomycin (all purchased from Life Technologies Gaithersburg, MD, USA). Transforming growth factor (TGF)-β2 and mouse T cell enrichment columns were purchased from R&D Systems (Minneapolis, MN, USA). Peptide IRBP1-20 (GPTHLFQPSLVLDMAKVLLD), representing residues 1–20 of human IRBP, was synthesized by Invitrogen (Carlsbad, CA, USA). Myelin basic protein (MBP), pertussis toxin (PTX) and incomplete Freund's adjuvant (IFA) were purchased from Sigma Life Sciences (St Louis, MO, USA). Heat-inactivated desiccated *Mycobacterium tuberculosis* (H37 RA) was purchased from Difco laboratories (Detroit, MI, USA). Tropicacyl® (tropicamide ophthalmic solution 1%) and phenylephrine hydrochloride ophthalmic solutions 2·5% were both purchased from Akorn Inc. (Lake Forest, IL, USA). Monoclonal antibodies for flow cytometry such as Fc block (anti-2.4G2), forkhead box protein 3 (FoxP3) fluorescein isothiocyanate (FITC) (clone-FJK-16s), CD8 phycoerythrin (PE) (clone LY3), CD4 PE (clone GK1-5), anti CD253 TNF-related apoptosis-inducing ligand (TRAIL) PE (clone N2B2), F4/80 FITC (clone BM8), CD40 PE (clone IC10), as well as mouse interferon (IFN)-γ and mouse interleukin (IL)-17A enzyme-linked immunosorbent assay (ELISA) kits were purchased from eBioscience Inc. (San Diego, CA, USA). Mouse CD8^+^ and CD4^+^ T cell isolation kits were purchased from Miltenyi Biotech (Auburn, CA, USA).

### Flow cytometry and cell sorting

Splenic cells that were analysed by flow cytometry were stained in the presence of a saturated concentration of Fc block (blocks FcRγ II/III). Cells (1 × 10^6^) were stained with the monoclonal antibodies using concentrations recommended by the manufacturer. Stained cells were analysed on a BD LSRII Flow analyser (BD Biosciences, San Diego, CA, USA). For sorting TRAIL^+^ and TRAIL^–^ populations, enriched CD8^+^ T cells were passed through a MoFlo Cell Sorter (Cytomation, Inc., Fort Collins, CO, USA).

### Induction of EAU

EAU was induced by modification of methods reported [7]. Briefly, donor B6 mice were immunized subcutaneously (s.c.) with 100 μl of an emulsion (1:1) of phosphate-buffered saline (PBS) and IFA containing 200 μg of IRBP_1–20_ and 500 μg of *M. tuberculosis* H37RA (Difco Laboratories). A single dose of PTX (200 ng) was injected intraperitoneally (i.p.) on the same day. The lymphocytes from draining lymph nodes and spleens of the immunized donor mice were collected on day 12 and activated in culture with 30 μg/ml of IRBP_1–20_ for 48 h, after which the non-adherent cells were collected, washed and injected [5 × 10^6^ cells/0·1 ml PBS/intravenously (i.v.)] into recipient B6 mice to induce EAU.

### Scoring of EAU

The ocular fundus of the mouse eyes was examined by slit lamp two times a week for clinical signs of EAU. Pupils were dilated using Tropicacyl® and phenylephrine hydrochloride ophthalmic solutions. The severity of inflammation was clinically graded on a scale of 1–5, as described previously [12,13]. In brief, a grade of 1 or less was considered as a negative score. Briefly, 0 = no inflammation; 1 = focal vasculitis ≤5 spots or soft exudates ≤5; 2 = linear vasculitis or spotted exudates ≤50% of the retina; 3 = linear vasculitis or spotted exudates ≥50% of the retina; 4 = retinal haemorrhage or severe exudates and vasculitis; and 5 = exudative retinal detachment or subretinal (or vitreous) haemorrhage. A mouse was considered to have uveitis if at least one of its eyes had a score of above 1 or more. The severity of uveitis is represented as the highest clinical score achieved by either eye in a mouse over the 25 days of the clinical disease. The clinical symptoms of EAU post-transfer of IRBP immune cells are less severe than the clinical symptoms of EAU induced by traditional immunization (includes CFA and pertussis toxin).

### Histopathological evaluation

Whole eyes were collected at the peak of the clinical response (between 21–23 days after induction of EAU by adoptive transfer of IRBP immune cells), immersed in 10% formaldehyde and stored until processed. Fixed and dehydrated tissues were embedded in methacrylate, and 5-μm sections were cut through the papillary–optic nerve plane and stained with haematoxylin and eosin (H&E). The presence or absence of disease was evaluated in a blinded fashion by examining six sections cut at different levels for each eye.

### Preparation of TolAPC

**Figure 1:**
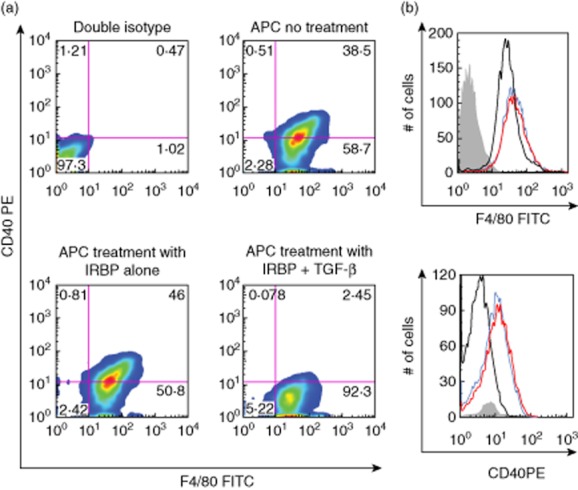
Flow analysis of CD40 expression. (a) Antigen-presenting cells (APC) were treated with transforming growth factor (TGF)-β2 and interphotoreceptor retinoid-binding protein (IRBP) overnight to produce tolerogenic antigen-presenting cells (TolAPC). F4/80 is plotted on the abscissa and CD40 on the ordinate. (b) Upper histograph of APC stained with F4/80 after various treatments. Lower histograph of APC gated for F4/80 fluorescein isothiocyanate (FITC)-positive cells and analysed for CD40 phycoerythrin (PE). TolAPC (black); APC without treatment (blue); APC pulsed with IRBP (red). Shaded graph represents the isotype control. Representative of two experiments.

TolAPC were prepared by a modification of methods reported [14–17]. Briefly, thioglycolate-elicited PEC was cultured overnight in SFM with TGF-β (5 ng/ml) and antigen [IRBP_1–20_ (50 μg/ml), retinal extract (100 μg/ml), corneal extract (100 μg/ml) or MBP (100 μg/ml)]. After incubation, the culture media was replaced with cold (4°C) PBS for 10 min, and the APC were removed by gently scraping the Petri dish with a rubber policeman. To verify that TolAPC were generated, the APC were analysed by flow cytometry for expression of CD40 and F4/80. CD40, a co-stimulatory molecule for immune activation, was down-regulated but F4/80, a surface marker associated with anterior chamber (a.c.)-associated immune deviation (ACAID) TolAPC [18], was increased (Fig. 1). Recovered APC were suspended in PBS (10^7^ cells/ml). Each recipient mouse was inoculated (i.v.) with 100 μl of cell suspension (10^6^ cells) 7 days after induction of EAU.

### Preparation of T cells from spleens for treatment of EAU mice

Because, in most experimental animal groups, the EAU peak clinical response subsided by 24 days, we collected cells between 21 and 23 days post-initiation of EAU. Spleens were dissociated individually into single-cell suspensions and labelled as (i) EAU untreated or (ii) EAU-treated. Dissociated spleen cells were passed through T cell enrichment columns (R&D Systems). The T cell samples (5 × 10^6^/100 μl PBS) were separated further into CD8-or CD4-positive populations, using magnetic isolation kits on fluorescence-activated cell sorting before being injected into new EAU mice. Cells from our donor mouse were injected (i.v.) into one recipient EAU mouse.

### *In-vitro* correlate for ACAID

It has been shown previously that induction of ACAID by antigen inoculation into the a.c. could be bypassed by injecting TolAPC i.v [16]. Furthermore, it is known that *in-vitro* ACAID cultures where the TolAPC is co-cultured (5–7 days) with spleen cells will generate both CD4^+^ and CD8^+^ T_reg_ cells. Thus, TolAPC generates T_reg_ cells *in vitro* and *in vivo* [19]. To generate sufficient numbers of the T_reg_ cells we used *in-vitro* cultures [16]. IRBP-pulsed TolAPC were incubated with non-adherent spleen cells for 7 days. The non-adherent cells were harvested and enriched for CD8^+^ T cells using the magnetic affinity cell sorting (MACS®) cell separation system following the manufacturer's instructions (Miltenyi Biotech). The purity of the separated cells was checked using flow cytometry PE-conjugated CD8 monoclonal antibody.

### Assay of *in-vitro* T helper type 1 (Th1) and Th17-related cytokine production

Spleens were removed from each group of TolAPC or untreated APC-treated mice 22 days after induction of EAU. Cells were seeded in flat-bottomed, 12-well tissue culture plates at a density of 6 × 10^6^ cells per well in 2 ml of the culture medium RPMI-1640 supplemented with 10% fetal calf serum, 2-mM of L-glutamine, 1-mM of sodium pyruvate and antibiotics in the presence of hIRBP_1–20_ peptide (50 μg/ml) and then cultured for 48 h. To assess cytokine production, cell-free supernatants were collected at 48 h and assayed for IFN-γ and IL-17 by using a mouse IFN-γ and IL-17 ELISA (Ready-SET-Go! Kit; eBioscience, Inc.).

### Local adoptive transfer (LAT) assay [20]

The LAT assay is commonly used to test the efferent suppressor activity of a population of cells [21]. The concept is to inject (intradermally) a mixture of immune cells with antigen into the ear pinnae of a naive mouse. The mouse serves as a test tube for the immune response; the swelling of the ear is evidence of a delayed hypersensitivity response induced by the injected immune cells and antigen. CD8^+^ T_reg_ cells (5 × 10^5^ cells) were mixed with spleen cells (5 × 10^5^ cells) from mice immunized with IRBP and CFA 7 days previously and antigen was injected (10 μl/injection) into the ear pinnae of B6 mice. The ear thickness was measured with an engineer's micrometer before and compared to ear thickness 24 h after injection.

### Preparation of retinal extract

The eyes were enucleated from euthanized mice. The eyeballs were cut at the equator around the ora serrata, and the posterior pole of the eyes was separated from the anterior pole and lens. The retina, consisting of the neural retina and the retinal pigment epithelial cells, was extracted from the posterior pole. The extract from one retina was placed in 500 μ of RPMI on ice (1 min) and sonicated briefly three times for 7 s at a probe intensity of 7 (Microson™ XL2000 Ultrasonic liquid processor; Qsonica, LLC, Newton, CT, USA). After removal of the insoluble material by centrifugation (200 ***g*** for 5 min), the protein concentration of the retinal extract was measured at 280 nm on an ND-1000 spectrophotometer, and adjusted to approximately 4 mg/ml. The retinal extract (100 μg/ml) was used as antigen to pulse the TolAPC.

### Preparation of corneal extract

A 1·5-mm full-thickness cornea button was trephined (under the hydration of PBS) from euthanized mouse tissue, and sonicated in RPMI (100 μl) on ice to prepare a homogeneous solution. The protein concentration of the corneal extract from one corneal button was approximately 2 mg/ml. A dilution of the corneal extract (100 μg/ml) was used to pulse the TolAPC.

### Statistical analysis

All statistical analyses were performed using PRISM™ software. Statistical differences in the incidence of uveitis and peak clinical scores between controls *versus* experimental groups were determined by non-parametric Mann–Whitney *U*-tests. In some experiments statistical differences between the course of the EAU (area under the curve) between groups were also compared using non-parametric Mann–Whitney *U*-tests. Statistical differences between cytokine production in control and treated EAU in ELISA assays were determined using a one-tailed Student's *t*-test. Differences were considered significant at *P* ≤ 0·05.

## Results

### IRBP-pulsed TolAPC suppress pre-existing EAU

**Figure 2:**
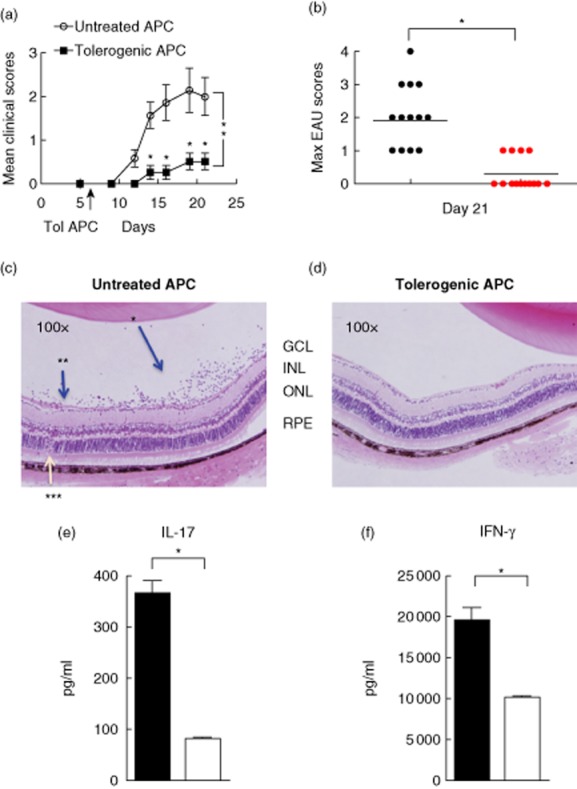
Effect of tolerogenic antigen-presenting cells (TolAPC) on clinical course of experimental autoimmune uveitis (EAU). (a) Average clinical score over time of EAU in mice with and without TolAPC treatment. EAU was induced by adoptive transfer of interphotoreceptor retinoid-binding protein (IRBP)-sensitized enriched T cells into C57BL/6 mice. A week later, TolAPC (red *n* = 14) or untreated antigen-presenting cells (APC) (black *n* = 14) were injected intravenously (i.v., 10^6^ cells/mouse) into EAU mice. Data shown are the mean clinical score (ordinate) of each experiment group over time (abscissa), and are the sum of two independent experiments. Comparison of (the course of the clinical symptoms) untreated EAU mice *versus* TolAPC-treated mice shows a significant difference (*P* ≤ 0·05) and is indicated. (b) Scatterplot shows peak scores on day 21 of individual EAU mice were given TolAPC (red) or not (black). The peak clinical scores over time of TolAPC-treated EAU mice are significantly lower than the peak scores over time of the untreated EAU mice. *Indicates a significant difference (*P* ≤ 0·05). (c,d) Photomicrographs of haematoxylin and eosin (H&E)-stained retinal tissue. Representative photomicrographs paraffin-fixed H&E stained slides of the retina of (c) EAU mice that received untreated APC (*leucocytes in vitreous cavity; **swelling; ***retinal fold) and (d) EAU mice that received TolAPC days post-initiation of EAU. Retinal pigment epithelium (RPE), outer nuclear layer (ONL), inner nuclear layer (INL), ganglion cell layer (GCL). (e,f) Enzyme-linked immunosorbent assay (ELISA) analysis of inflammatory cytokines in spleen cells harvested at day 23 of EAU mice that received either TolAPC or spleen cells were restimulated with antigen with serum-free media for 48 h prior to collecting the supernatants for analyses. Bar graphs showing (e) interleukin (IL)-17 and (f) interferon (IFN)-γ production. Spleen cells (from three separate mice) were harvested from APC-(solid bar) or TolAPC-treated EAU mice (three per group) co-cultured with antigen (IRBP 50 ug/ml) (open bar). Supernatants from duplicate cultures were harvested 48 h after restimulation with IRBP for ELISA analysis. An asterisk (*) indicates a significant difference (*P* ≤ 0·05).

TolAPC were generated by culturing thioglycolate-elicited peritoneal exudate cells (PEC) in serum-free medium (SFM) in the presence of IRBP_1–20_ and TGF-β2. Untreated APC were cultured in SFM only, without IRBP and TGF-β2. The TolAPC or untreated APC were injected (i.v. 10^6^ cells/mouse) into EAU mice. EAU was induced by IRBP-sensitized cells that were transferred adoptively, as described in Materials and methods. Mice with EAU that were treated with TolAPC exhibited a delay in disease onset and a significantly (*P* ≤ 0·001) lower peak EAU score over time (Fig. 2a,b). The mice that received untreated APC treatment had a mean clinical severity score of 2 ± 0·26, while the mice that received TolAPC treatment had a mean clinical severity score of 0·64 ± 0·13 (*P* ≤ 0·05) (Table 1). In addition, examination of haematoxylin and eosin (H&E)-stained paraffin-fixed slides revealed that retinal sections of eyes from EAU mice that received TolAPC showed a reduced cell infiltration into the vitreous cavity and their retinal layer structures lacked the retinal folds and vascular swelling observed in the untreated mice (Fig. 2c,d). The fact that the mice given TolAPC had peak clinical scores of 1 or lower (mild or no uveitis) supported the postulate that the TolAPC induced suppression of the clinical symptoms of EAU. Thus, treatment of EAU mice with IRBP-pulsed TolAPC a week after induction of the EAU delayed its onset and suppressed the subsequent development and severity of EAU (Table 1).

**Table 1:** Incidence of uveitis and peak disease score of mice after untreated antigen-presenting cells (APC) *versus* tolerogenic antigen-presenting cell (TolAPC) treatment.

Treatment of EAU mice	Incidence of mice with clinical score ≥2	Peak disease score ± s.e.m.
Untreated APC	9/14	2·0 ± 0·026
Tolerogenic APC	0/14*	0·64 ± 0·13*

Scores are from the experimental and control mice used for Fig. 2.

* Indicates a significant difference (*P* ≤ 0·05) between untreated and TolAPC-treated mice. EAU: experimental autoimmune uveitis; s.e.m.: standard error of the mean.

### TolAPC treatment down-regulates the production of EAU-associated inflammatory cytokines

To examine the effect of TolAPC treatment on the production of inflammatory cytokines, IFN-γ and IL-17 [11] we collected non-adherent cells from the spleens of EAU mice that received TolAPC or untreated APC. The spleen cells were restimulated with IRBP antigen for 48 h *in vitro* and assayed for the production of IFN-γ and IL-17. Spleen cells derived from TolAPC-treated EAU mice produced significantly (*P* ≤ 0·05) less IFN-γ (Th1) and IL-17 (Th17) than EAU mice receiving untreated APC (Fig. 2e,f). Thus, TolAPC, but not APC, treatment resulted in suppression of the inflammatory cytokine response in EAU mice.

### Ability of retinal extract-*versus* IRBP-pulsed TolAPC to suppress EAU

**Figure 3:**
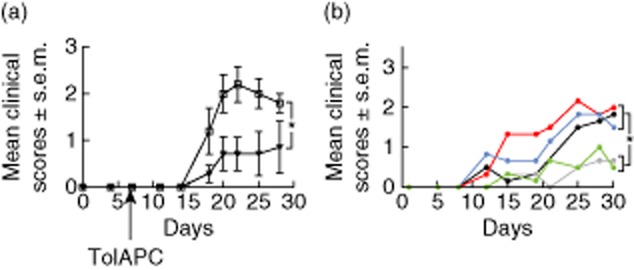
Effect of retinal antigen-pulsed tolerogenic antigen-presenting cells (TolAPC) on clinical course of experimental autoimmune uveitis (EAU) induced with interphotoreceptor retinoid-binding protein (IRBP). (a) Comparison of clinical scores of EAU mice treated with retinal extract-pulsed TolAPC (filled triangles, *n* = 7) or EAU mice not treated (open squares, *n* = 7). Data are shown as mean clinical EAU score ± standard error of the mean (s.e.m.) (ordinate) over time (abscissa). The retinal extract-pulsed TolAPC-treated mice show a significant (*P* ≤ 0·05) decrease in EAU clinical score over time compared to scores of EAU in untreated mice. (b) Antigen specificity of TolAPC-induced suppression. The EAU mice were treated with each type of TolAPC, 7 days post-induction of EAU (Materials and methods). Line graph of response of EAU mice to TolAPC pulsed with indicated antigens. The transferred antigen-presenting cells (APC) were not pulsed with antigen (black line, *n* = 15) or were treated with transforming growth factor (TGF)-β and pulsed with corneal extract (red line, *n* = 7), myelin basic protein (MBP) (blue line, *n* = 6), IRBP_(1–20)_ (grey line, *n* = 14) or retinal extract (green line, *n* = 16). Data shown are mean clinical score (ordinate) over time (abscissa). An asterisk (*) indicates a significant difference between the areas under the curves. Statistics were performed using Prism software (Materials and methods).

Although the retinal antigens that induce the EAU in the mice are known, the target antigens in human uveitis remain obscure. Here, we tested if retinal protein extract (containing IRBP and other retinal antigens) would provide the relevant antigens for producing the TolAPC that were effective in this model of suppression. In this experiment EAU was induced by injecting IRBP-specific cells as before, but the TolAPC were made by incubation with TGF-β and IRBP or mouse retinal extract. The retinal antigen-pulsed TolAPC were then injected (i.v.) into the EAU mice, 7 days post-induction with the IRBP-specific cells. As before, mice were monitored and clinical symptoms were scored every 3–30 days. We observed that the retinal extract (but not corneal extract, control)-pulsed TolAPC were as effective as IRBP_1–20_-pulsed TolAPC in reducing the clinical symptoms of EAU (Fig. 3a). Furthermore, if the TolAPC were pulsed with the irrelevant antigen MBP, they were not able to establish suppression of EAU (Fig. 3b). Therefore, it is possible to produce EAU-specific TolAPC when the TolAPC are pulsed with retina extract.

### TolAPC induce T_reg_ cells in the spleens in host EAU mice

**Figure 4:**
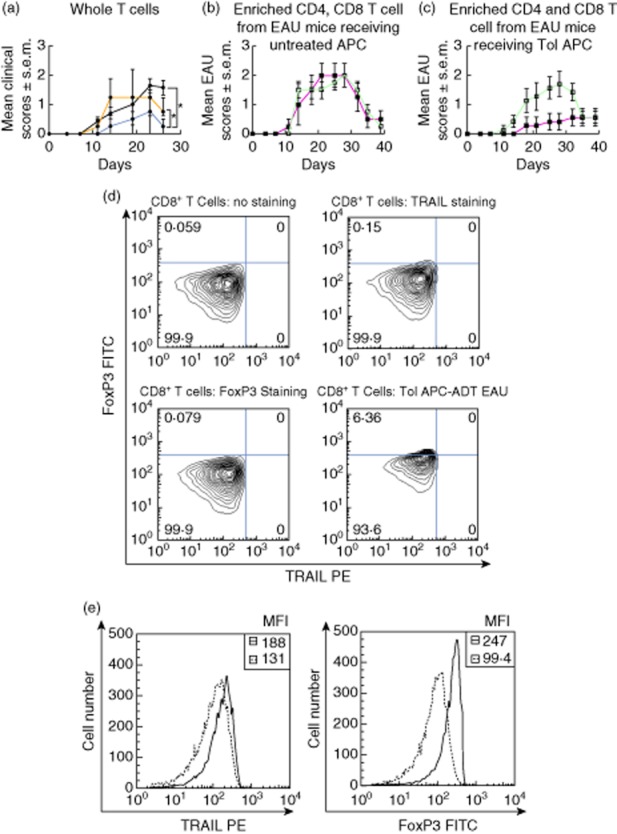
The regulatory effects of CD4^+^ or CD8^+^ T cells from tolerogenic antigen-presenting cells (TolAPC)-treated experimental autoimmune uveitis (EAU) mice. The line graph represents progression of mean clinical scores of EAU over time. The ordinate = mean EAU clinical scores; abscissa = days post-induction of EAU. Whole T cells, CD4^+^ or CD8^+^ T cells were collected from the spleens of mice that were either TolAPC or antigen-presenting cells (APC). Enriched T cells were collected at the peak of clinical symptoms and transferred to a new group of EAU mice to test for their suppressor function. (a) Clinical course of EAU in mice receiving no T cells (*n* = 14, black line); clinical course of EAU in mice receiving T cells harvested from the EAU mice were injected with APC (*n* = 4, orange line); clinical course of EAU in mice receiving T cells harvested from the EAU mice that were injected with TolAPC (*n* = 4, blue line). (b) Effects on EAU of mice receiving enriched T cells (CD8^+^ T red line; CD4^+^ T green line) harvested from the EAU mice were injected with APC. (c) Effects on EAU of mice receiving enriched T cells (CD8^+^ T red line; CD4^+^ T green line) from EAU mice that were injected with TolAPC (*n* = 7 per group). Each recipient mouse received one donor equivalent. *Significant difference (*P* ≤ 0·05) in overall severity of the disease over time between two indicated experimental groups. (d) Representative flow cytometry analysis of CD8^+^ T cells handled in the same manner as the CD8^+^ T cells in (c). Upper left block shows resting CD8^+^ T cells with no staining; upper right block are resting CD8^+^ T cells stained with tumour necrosis factor (TNF)-related apoptosis-inducing ligand (TRAIL) antibody only; lower left block shows resting CD8^+^ T cells stained with forkhead box protein 3 (FoxP3) antibody only; and lower right shows the CD8^+^ T cells harvested from the TolAPC-treated mice used in (c) (red line) stained from FoxP3 FITC (ordinate) and TRAIL phycoerythrin (PE) (abscissa) (e) Mean fluorescence intensity (MFI) analyses of CD8^+^ T cells from TolAPC-treated EAU mice (solid line) compared to staining on CD8^+^ T cells from EAU mice. The abscissa represents the intensity of the fluorescence for the indicated antibody; the ordinate shows the cell number; insert box gives the actual reading for the MFI. Dashed line (—) APC-treated EAU CD8^+^ T cells; solid line (—) TolAPC-treated EAU CD8^+^ T cells. The left block shows that there is a little or no increase in fluorescence intensity staining for TRAIL in the two types of CD8^+^ T cells compared. The right block shows a major shift in staining intensity for the MFI of FoxP3 staining and CD8^+^ T cells from the TolAPC-treated EAU mice.

Having shown that TolAPC treatment suppressed the production of inflammatory cytokines and reduced the clinical symptoms of EAU, we next analysed the cellular mechanisms that might be responsible for the suppression. It is known that tolerance induced by antigens a.c.-inoculated or by TolAPC-inoculated i.v. induce T_reg_ cells that can transfer tolerance. To test for the presence of T_reg_ cells, T cells were enriched from spleens harvested (day 21) from individual EAU mice treated with TolAPC, restimulated with IRBP *in vitro* for 48 h prior to transferring (5 × 10^6^, 100 μl) to syngeneic recipients in which EAU had been induced 7 days previously. Control groups of mice received either restimulated enriched T cells from EAU mice that were injected with untreated APC or were not injected with APC. EAU mice receiving T cells from mice treated with TolAPC exhibited a delayed onset of their EAU symptoms with less severity compared with the EAU mice that received cells from the control groups of mice (*P* ≤ 0·05) (Fig. 4a), suggesting that the suppression of the autoimmune inflammation was mediated by T_reg_ cells induced by the administered TolAPC.

### CD8^+^, but not CD4^+^, T_reg_ cells transfer suppression to EAU mice

Previous reports showed that either a.c. injection of antigen or i.v. transfer of *in-vitro*-generated TolAPC induced two types of T_reg_ cells: afferent CD4^+^ T_reg_ and efferent CD8^+^ T_reg_ cells. Both the CD4^+^ and the CD8^+^ T_reg_ cells have been characterized [22,23]. To determine which type of T_reg_ cells transferred the tolerance to EAU mice, the experiments were repeated. Spleen cells were dissociated from spleens harvested from EAU mice at 21 days and restimulated *in vitro* with IRBP. The spleen cells were collected from two groups of mice as follows: (i) individual EAU mice that received untreated APC (Fig. 4b) and (ii) individual EAU mice that received TolAPC (Fig. 4c). The spleen cells from each mouse were restimulated *in vitro* in separate cultures with IRBP (48 h), after which CD4^+^ T cell and CD8^+^ T cell populations were enriched by magnetic bead separation. We observed that transfer of efferent CD8^+^, but not the afferent CD4^+^ T cells, suppressed the clinical symptoms of EAU (Fig. 4a–c).

### Characterization of the T_reg_ cell

Both CD4^+^ and CD8^+^ T_reg_ cells that are generated after culturing spleen cells with TolAPC have been characterized by Keino and colleagues [22,23]. We analysed if the harvested CD8^+^ T cells expressed TRAIL. In brief, spleen cells were harvested from experimental mice that received TolAPC and had EAU scores of 1 or less. The dissociated spleen cells were restimulated with antigen for 48 h. Post-culturing, the non-adherent cells were collected, enriched by magnetic bead separation for CD8^+^ T cells, and an aliquot of the enriched CD8^+^ T cells was examined prior to transfer by flow cytometry for expression of TRAIL. Interestingly, the T_reg_ cells collected from TolAPC-treated EAU mice that transferred tolerance to a second set of EAU immunized mice were CD8^+^FoxP3^+^TRAIL^–^ (Fig. 4d). Analysis of the mean fluorescence intensity (MFI) of the CD8^+^ T cell populations analysed by flow cytometry showed little or no shift in TRAIL staining (Fig. 4e).

### Local adoptive transfer assay

Thus far, we show that *in-vitro*-generated TolAPC transferred to EAU mice suppress the clinical symptoms of EAU by generating CD8^+^TRAIL^–^ T_reg_ cells. To evaluate further the expression TRAIL on CD8^+^ T_reg_ cells, we generated CD8^+^ T_reg_ cells *in vitro* [24]. The suppressor function of the *in-vitro*-generated T_reg_ cells was evaluated in a local adoptive transfer assay (Materials and methods). Others have reported that T_reg_ cells are generated in cultures where TolAPC have similar characteristics to those generated *in vivo* [19,24–26].

**Figure 5:**
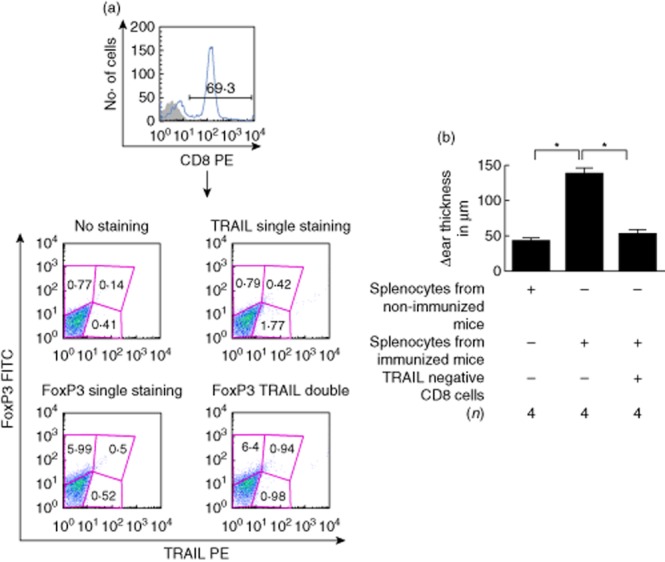
Effect of CD8^+^ tumour necrosis factor (TNF)-related apoptosis-inducing ligand (TRAIL)^–^ cells on suppression. (a) Flow cytometry gate used for analysis. Spleen cells were cultured with antigen-presenting cells (APC) treated with transforming growth factor (TGF)-β2 and interphotoreceptor retinoid-binding protein (IRBP). Seven days later the CD8^+^ T cells were enriched by magnetic beads and single-stained for CD8 (top panel). Lower panel: an aliquot of enriched CD8^+^ T cells was stained with TRAIL phycoerythrin (PE) (abscissa) and forkhead box protein 3 (FoxP3) (fluorescein isothiocyanate (FITC) (ordinate). (b) Local adoptive transfer assay using CD8^+^TRAIL^–^ T cells. The experiment was performed twice with similar results. *Significant difference (*P* ≤ 0·05).

After co-culturing F4/80^+^ TolAPC with spleen cells for 7 days, the non-adherent cells were harvested and the CD8^+^ T cells sorted into TRAIL-negative and-positive populations (Fig. 5a). The CD8^+^TRAIL^–^ T cells suppressed the response to IRBP in a LAT assay (Fig. 5b) (there were insufficient CD8^+^TRAIL^+^ cells to test their function). Flow analysis of the TRAIL^–^CD8^+^ T_reg_ cells confirmed that CD8^+^ T_reg_ cells expressed CD103 (data not shown) [22]. Thus, 7-day cultures of TolAPC with spleen cells generate CD8^+^CD103^+^FoxP3^+^TRAIL^–^ T_reg_ cells that are capable of suppressing efferent immune responses *in vivo*.

## Discussion

It is reasonable to think that if autoimmunity occurs in the eye, one or more mechanisms of immune privilege, immune regulation, must be compromised. ACAID is a model used to study ocular immune privilege *in vivo* and *in vitro* that generates peripheral antigen-specific T_reg_ cells [2,17,27,28]. As early as 1992, Streilein and colleagues reported that ACAID induction by intracameral inoculation of the immunizing ocular autoantigen prior to the induction of uveitis reduced the incidence of EAU in mice [29]. No ocular inflammation was observed in the group that received the retinal antigen via the a.c. prior to the induction of EAU, while 80% of mice that were injected a.c. with PBS developed uveitis. Here, we extended these studies and induced tolerance by transferring *in-vitro*-generated TolAPC to experimental mice after EAU was induced. APC become tolerogenic after exposure to immunosuppressive factors such as TGF-β2 and antigen *in vitro*, and have been shown to promote negative regulation of both Th1-and Th2-mediated inflammation in part by generating antigen-specific T_reg_ cells [25,27,30].

The transfer of antigen-pulsed TolAPC has been shown to induce tolerance in both naive and sensitized mice [15,31]. Furthermore, we have reported previously that the adoptive transfer of TolAPC successfully abrogated immune inflammation and clinical symptoms in mouse models for autoimmune pulmonary interstitial fibrosis [25], airway hypersensitivity hyper-reactivity [26] and experimental autoimmune encephalomyelitis (EAE) [32]. Here, we show that TolAPC pulsed with retinal antigen (IRBP or extract of retina) are capable of reducing the clinical symptoms and inflammatory cytokines in an adoptive transfer model of IRBP-induced EAU. Thus, depending on the frequency of the unknown target antigen in a retinal extract, this approach may provide a basis for a novel cell-based therapy using autologous cells for the treatment of ocular autoimmune diseases such as uveitis.

The TolAPC associated with eye-induced tolerance were first defined by their surface expression of F4/80 protein and were therefore thought to be macrophages [14,33–37]. However, once F4/80^+^ macrophages are mobile their appearance becomes dendritic [33]. Also, although F4/80 is expressed predominantly by macrophages, F4/80 is expressed by a subset of dendritic cells (DC) that is tolerogenic [38,39]. Importantly, F4/80 protein expression is necessary for the tolerogenic function of TolAPC [40]. Although F4/80 may not always distinguish macrophages from other APC, the inability to induce ACAID in op/op mice, a B6 mouse with a spontaneous mutation in the *csf* gene region resulting in a deficiency in some macrophage but not DC populations, supports the notion that the F4/80^+^ TolAPC are macrophages [41].

A variety of DC has been shown to be tolerogenic in experimental models [42–44]. While the majority opinion is that immature DC are tolerogenic [44–47], the use of immature DC for therapeutic reasons has limited potential because of the possibility that they could mature once exposed to the immune state of the recipient [48]. Other investigators contend that tolerogenic DC are semi-mature [49]; other reports show that a subtype of DC (plasmacytoid dendritic cells) has tolerogenic capabilities [50,51]. Thus, it becomes clear that several types of APC have the potential of becoming tolerogenic.

Our laboratory has been successful in generating TolAPC from thioglycolate-induced PEC and bone marrow-derived macrophage/DC [26] as well as macrophage hybridoma no. 59 [52,53]. We have also made TolAPC from enriched human DC isolated from human peripheral blood lymphocyte samples (unpublished data). Thus, our experience supports the idea that multiple types of APC have the potential of maturing into TolAPC if the critical components of TGF-β and antigen activation are present together. APC activated by antigen in the presence of TGF-β progress through distinct regulatory pathways, and subsequently express distinct regulatory markers. Therefore, we propose that immature macrophages/DC have the option to mature through multiple pathways into immune-activating or immune-regulating cells.

CD8^+^ T_reg_ cells were first identified in ACAID induction in the 1990s [54]. The CD8^+^ T_reg_ cells induced by antigen injection into the eye express CD103 and have a novel genetic pattern associated with their efferent suppressor function [22]. ACAID-induced CD8^+^ T_reg_ cells can suppress by secreting TGF-β2 [55]. One report suggests that a population of CD8^+^TRAIL^+^ T_reg_ cells develop in an extra-ocular environment post-antigen inoculation to the a.c. and mediate suppression [56]. In this paper, we analysed the CD8^+^ T cell population from the spleens of the TolAPC-treated and control-treated EAU mice for expressing TRAIL. We observed that CD8^+^ T cells harvested from TolAPC-treated EAU mice were able to transfer tolerance, but they were negative for TRAIL. Thus our data suggest that TolAPC injected into mice with EAU generate CD8^+^FoxP3^+^TRAIL^–^ T_reg_ cells can mediate and transfer efferent suppression of EAU. Apropos this observation are reports that support the idea that the CD8^+^ T_reg_ cells suppress by multiple mechanisms [57,58] and that the mechanisms are strain-dependent [59]. Reported studies show that C57BL/6 CD8^+^ T_reg_ cells express less TRAIL than BALB/c CD8^+^ T_reg_. Moreover, C57BL/6 CD8^+^ T_reg_ suppression is IL-35-, IL-10-dependent and BALB/c CD8^+^ T_reg_ suppression is TRAIL-dependent.

Consistent with the idea that CD8^+^ T_reg_ cells from different mouse strains use different methods to suppress is the posit that CD8^+^ T_reg_ suppressive mechanisms may vary with the substrain of mouse used. The C57BL/6 mouse is the most well-known inbred mouse strain and provides the genetic background for congenic and mutant mice. There are also a number of substrains derived from the founder B6 strain. The fact that the substrains express genetic and phenotypic variances is not always acknowledged in research papers. For instance, genotyping demonstrated genetic differences in the C57BL/6J and the C57BL/6N substrains at 11 single nucleotide polymorphism (SNP) loci [60]. The SNP pattern for the C57BL/6 mouse from NCI (the C57BL/6 CrSlc substrain) and the C57BL/6N substrain were the same [60]. Moreover, Mattapallil and colleagues identified the CRB1^rd8^ mutation of the retinal degeneration phenotype in the C57BL/6N but not the C57BL/6J substrain [61], and cautioned researchers that these mice provide the background from many genetically modified strains used in the study of the eye. Indeed, recent studies with C57BL/6 mice with the Crb1^rd8^ mutation with a CD11c expression of yellow fluorescent protein (eYFP) transgenic reporter show abnormal numbers of CD11c-positive cells in the retina of 8–10-week-old mice [62].

The studies reported here induced CD8^+^TRAIL^–^ T_reg_ cells in the C57BL/6J substrain, while the studies that induced the CD8^+^TRAIL^+^ T_reg_ cells post-antigen inoculation into the a.c. used C57 BL/6N mice homozygous for the Rd8 mutation [56]. Thus the markers expressed and the methods used to suppress immune responses by the ‘ACAID'-induced CD8^+^ T_reg_ cells may depend upon not only the strain of mouse used [59] but also the substrain used.

This is the first time that F4/80^+^ TolAPC has been used to treat existing inflammation in the eye. Others have shown that bone marrow-derived immature DC cultured in granulocyte–macrophage colony-stimulating factor (GM-CSF) and pulsed with antigen were able to inhibit EAU (induced with IRBP, CFA and PTX) if given before the induction of the uveitis [63]. Our experimental design differs from these and previous studies using ACAID mechanisms to suppress EAU [29], in that the antigen-pulsed, TGF-β2-treated TolAPC were given a week to 10 days after adoptively transferring EAU, suppressing an already established autoimmune response, supporting the possibility of the development of therapy for human autoimmune uveitis.

In summary, we show that TolAPC generated *ex vivo* by TGF-β2 treatment in the presence of EAU-inciting antigen (IRBP) or retinal antigen extract were able to modulate the clinical symptoms and inflammatory cytokines of IRBP-induced EAU in mice. Mechanistic studies showed that the efferent suppression could be transferred with CD8^+^FoxP3^+^TRAIL^–^ T_reg_ cells. Together, these observations raise the possibility that the clinical symptoms of human uveitis might be relieved by therapy that uses target tissue extract instead of a specific antigen (currently unknown) to generate TolAPC from the patients' own cells for autologous transfer.
